# Clinical significance of biologic width in dentistry

**DOI:** 10.6026/973206300223692

**Published:** 2026-06-30

**Authors:** Krutika Sangani, Bindiya Trambadiya Bhimani, Ana Cristina Garcia, Kelly J. Pedraza Corrales, Zankhana Bhagat, Achalben Patel

**Affiliations:** 1 Department of Dentistry, Pacific Dental College and Hospital, Debari, Rajasthan, India; 2 Department of Dentistry, Ahmedabad Dental College and Hospital, Ranchodpura, Ahmedabad, India; 3 Department of Dentistry, Doctor of Dental Surgery, Universidad Santa Maria, Caracas, Venezuela, South America; 4 Department of Dentistry, Doctor of Dental Surgery, Specialist in Periodontics, Universidad Antonio Narino, Bogota, Colombia; 5 Department of Dentistry, K.M.Shah Dental College and Hospital, Piparia, Vadodara, Gujarat, India; 6 Department of Dentistry, Faculty of Dental Science, Nadiad, Gujarat, India

**Keywords:** Supracrestal tissue attachment, periodontium, invasion of biologic width, margin placement in restorative and implant dentistry, digital assessment tools for periodontal disease

## Abstract

Biologic width (BW) is the distance between the junctional epithelium and the supracrestal connective tissue above the alveolar crest, typically measuring approximately 2.04 mm. It is crucial for the health of periodontal, gingival, and alveolar bone tissues this creates a seal around the tooth's neck to prevent periodontal disease and gingival inflammation. Violation of BW can create a myriad of complications, like gingival inflammation, attachment loss, and bone loss. The article further discusses restorative and implant margin placement strategies, as well as surgical interventions such as crown lengthening, which help preserve BW. New digital tools provide a more precise assessment of BW in dentistry.

## Background:

The concept of biologic width (BW) has progressively assumed a central role, described as the histological space occupied by the supracrestal soft tissues that form the dentogingival junction (DGJ) [1]. Gargiulo *et al.* established the anatomical and physiological foundations of the DGJ through a pioneering histometric study that precisely defined the mean dimensions of its components: gingival sulcus depth (0.69 mm), junctional epithelium (0.97 mm) and supracrestal connective tissue attachment (1.07 mm), yielding a total biological width of approximately 2.04 mm [1]. Later, Ingber *et al.* introduced the concept of biologic width as a key reference in prosthetic and periodontal treatment planning. The integrity of this dimension is crucial in preventing inflammation, loss of attachment and bone resorption [2]. Several studies have demonstrated that violating this area by placing subgingival restoration margins without respecting the biologic width triggers chronic inflammatory responses and periodontal complications, many of which are irreversible [3]. Likewise, the literature has explored surgical strategies, such as crown lengthening via gingivectomy or osteotomy, procedures that enable the restoration of function, esthetics and periodontal health without compromising long-term prosthetic stability [4]. Therefore, it is important to establish updated and standardized criteria for evaluation, preservation and surgical management of biologic width, especially in light of the increasing adoption of minimally invasive techniques and increasing focus on aesthetic outcomes [5].

## Methods of assessing biological width:

## Clinical assessment:

While probing restoration margins, if the patient complains of pain, it indicates that the margin has extended into the attachment, violating the biological width. Clinical signs of biological width violation are clinical attachment loss, alveolar bone loss, gingival recession, pocket formation, chronic gingival inflammation around the restoration, localized gingival enlargement with minimal bone loss and bleeding upon probing. Often, overgrowths are seen when placing subgingival margins or when altering passive eruption [6].

## Bone sounding:

Bone sounding determines biological width by probing under local anesthesia until the bone is reached. From this calculated measurement, subtract the sulcus depth. If the difference in value is less than 2 mm at one or more sites, biological width violation is confirmed. Performing this test on multiple teeth with healthy gingiva will reduce site variability and ensure the accuracy of the results [6].

## Radiographic evaluation:

Radiographic interpretation helps detect biological violations at interproximal sites. Mesiofacial and distofacial line angles are less reliable due to the superimposition of the tooth [7]. To overcome this issue, Sushama and Gouri developed a new parallel profile radiographic (PPR) technique that accurately measures dentogingival unit (DGU) dimensions. This innovative method is simple, reproducible, non-invasive and capable of assessing the length and thickness of the DGU [8].

## Biologic width and periodontal health:

Biologic width is a specific concept that describes the dimensional relationships among epithelial attachment, sulcus depth, connective tissue attachment and the alveolar crest. This natural seal is essential for maintaining periodontal health and its disruption can lead to periodontal damage. An adequate understanding of biologic width is essential to ensure the form, function and esthetics of the dentition [9]. Encroachment into this dimension leads to chronic inflammation, clinical attachment loss and alveolar bone resorption. Clinical evidence indicates that placement of sub gingival restorative margins that violate the BW often leads to long-term complications such as gingival inflammation and recession. Conversely, maintaining an appropriate distance between the restorative margin and the alveolar bone crest helps to preserve periodontal health and ensure restorative longevity [10]. When the biological width is insufficient for restorative procedures, increasing this physiologic dimension surgically should be considered before restorative treatment begins. Procedures such as surgical crown lengthening or orthodontic extrusion are commonly recommended to create adequate space between the restorative margin and the alveolar bone, thereby preventing biologic width violation and future periodontal breakdown [11].

## Biologic width considerations in restorative dentistry:

According to Ingber *et al.* (1977), at least a minimum of 3 mm of space between the alveolar bone crest and the restorative margin of the tooth is necessary for better tissue healing and functional restoration of the tooth [2]. In restorative dentistry, there are three options for margin placement: supragingival, equigingival and subgingival.

## Supragingival margin:

When placed above the gumline, it has minimal effect on the periodontal tissue. It is easy to clean and adjust and less irritating to the gingival tissue. Dentists used it in non-cosmetic areas because old materials didn't blend well with natural tooth color. However, with the advent of new materials such as resin cements, more translucent restorations and modern adhesive techniques, it is now possible to place a supragingival margin in esthetic zones [10].

## Equigingival margin:

It is more prone to plaque accumulation than supragingival and subgingival margins, resulting in gingival inflammation. Even a minor gum recession can expose the restorative margins, resulting in an unattractive appearance. With modern dentistry, restorative margins merge with natural tooth structure, creating a smooth, finished margin interface at the gumline [12].

## Subgingival margin:

Factors such as tooth defects, sealing the tooth and restoring in esthetic areas and deep caries often require subgingival restorative margins to be placed beneath the gingival crest. This biologic width encroachment for better retention leads to iatrogenic periodontal diseases and early restoration loss. When dentists place a subgingival margin too close to the gingival crest, it can cause gingival inflammation. The margin can encroach on the gingival tissue, worsening the condition due to the patient's inability to clean the area effectively. The body tends to re-establish space between the alveolar bone and the restorative margin to allow tissue reattachment. This would happen more commonly in areas where the alveolar bone is thin. Typically, when deep margins are placed, the bone level remains constant; however, gingival inflammation arises and persists around the restored tooth [13]. Several investigators have reported that subgingival margin placement is associated with qualitative and quantitative shifts in the subgingival microbial system, increased gingival and plaque indices, increased pocket depth and gingival recession [14].

## Biologic width in implant dentistry:

Biologic width forms a protective barrier between a dental implant and the surrounding bone, which guards against infection and inflammation. It spans from the top of the gum tissue around the implant (peri-implant mucosa) to the point where the bone connects with the implant surface (bone-to-implant contact or BIC). Like natural teeth, this width typically measures about 3-4 mm. A restoration margin placed too close to the bone can disrupt this protective zone, potentially leading to persistent inflammation, bone resorption and soft tissue recession [15].

## Components of implant biologic width:

According to a comprehensive review, the biologic width around dental implants is composed of three distinct zones: sulcular epithelium, junctional epithelium and connective tissue. The junctional epithelium develops from different sources in implants and teeth, oral epithelium in implants and reduced enamel epithelium in teeth, yet the resulting tissue exhibits comparable morphology [16].

## Adaptation to prosthetics:

If a restoration encroaches on the biologic width, bone may resorb to restore it. Peri-implant mucosa forms a cuff-like barrier similar to gingiva, but the biologic width around implants (~3.8 mm) is slightly greater than around natural teeth (~3.2 mm), due to denser, scar-like connective tissue versus the organized periodontal attachment of teeth [17].

## Importance of biologic width around implants:

Maintaining this dimension is essential for peri-implant tissue health, as it prevents bacterial invasion, inflammation and subsequent bone loss. Proper establishment of the biologic width contributes to long-term implant stability, predictable osseointegration and favorable esthetic outcomes. Moreover, respecting this soft tissue seal is crucial during surgical procedures, restorative placement and maintenance therapy, as violations of the biologic width have been associated with peri-implant mucositis, peri-implantitis and compromised restoration longevity [18].

## Clinical implications and future directions:

The biologic width is a protective space that maintains the health of the gums and bone surrounding teeth and implants. Preserving the BW is essential in restorative dentistry, as it helps maintain gingival health and prevents long-term complications [9].

## Periodontal perspective:

When a filling or crown margin is placed too deeply and violates the BW, it can lead to chronic gingival inflammation, attachment loss and bone resorption, ultimately compromising esthetics and function. On the other hand, several procedures can help re-establish the BW. Orthodontic extrusion can move the restoration margin away from the bone by extruding the tooth. Alternatively, a dentist can perform crown lengthening surgery to remove bone and soft tissue, thereby exposing additional coronal tooth structure. When multiple teeth in a quadrant need crown lengthening, the dentist can perform an apically repositioned flap surgery. However, dentists do not recommend this procedure for single-tooth crown lengthening in the esthetic zone [19].

## Restorative dentistry:

Maintaining the natural space between the gingiva and the alveolar bone is critical for restorative success and periodontal health. Studies have shown that the ideal restoration should use a supra-gingival margin whenever possible. With advances in adhesive restorative materials, the dentist can now place supra-gingival margins even in esthetic areas and should prefer them whenever feasible [19].

## Implant dentistry:

Peri-implant soft-tissue healing, including the formation of a physiologic peri-implant biologic width, is essential for long-term implant function. Establishment of a stable soft-tissue seal requires a minimum biologic width; when this dimension is inadequate, crestal bone resorption may occur to facilitate biologic width formation [20]. Soft tissue attachment around implants resembles that of natural teeth but differs in certain aspects. The junctional epithelium is usually thinner and shorter because implants lack cementum and the parallel connective tissue fibers that run along the implant surface and they have a narrow avascular zone. The biological width around implants is essential for maintaining healthy and stable soft tissues. Factors such as implant design, the quality of surrounding tissue and adjacent teeth influence its dimensions. One-piece implants may better replicate the natural BW. Platform switching alters the implant-abutment connection, which can help preserve bone and maintain soft tissue stability. These features are essential for esthetic restorations, where long-term success depends on stable and healthy soft tissue [21]. Implant design, particularly the distinction between one- and two-piece systems, influences biologic width formation and peri-implant tissue health. Two-piece implants may elicit adaptive changes in biologic width and marginal bone levels due to the implant-abutment microgap, whereas one-piece implants tend to maintain a biologic width and gingival profile comparable to natural teeth. Although long-term soft-tissue stability appears similar between both designs, implant design remains a critical factor in the initial tissue response and long-term maintenance of biologic width [22].

## Future directions:

Digital tools are enhancing BW assessment by allowing clinicians to visualize the distance between the gingival margin and the alveolar crest, as well as soft tissue thickness, in a non-invasive way. Intraoral scanners, with their increasing accuracy, facilitate digital workflows that can track soft tissue changes and enable more predictable preparations that respect the BW [15]. As soft tissue thickness and the dentogingival complex vary by patient and tooth, future care will likely shift toward personalized BW profiles rather than relying on a single, fixed guideline. However, essential research needs remain multicenter, long-term trials that validate digital measurements against clinical outcomes and the development of consensus-based protocols that integrate prosthodontics, periodontics and implantology into unified guidelines [21]. The present findings emphasize the clinical importance of biologic width, currently referred to as supracrestal tissue attachment, in maintaining periodontal health and restorative success. Violation of this anatomical dimension may result in chronic gingival inflammation, attachment loss, pocket formation, and alveolar bone resorption. Similar observations have been reported by Padilla-Avalos *et al.* [23], Al Machot *et al.* [24], and Alam *et al.* [25], who highlighted the significance of preserving biologic width during restorative and periodontal procedures. Proper assessment and management of biologic width are therefore essential to achieve long-term functional and esthetic outcomes in dental practice.

## Conclusion:

Biologic width is the foundation for long-term success in periodontal, restorative and implant therapy. Biologic width violations can be corrected using surgical crown lengthening or orthodontic techniques. The coming decade will likely see advances in digital tools and personalized approaches that will further protect and optimize this critical zone.
